# Advantages and challenges of cardiac magnetic resonance spectroscopy at 3Tesla - applications to studies of cardiac steatosis in obesity and type 2 diabetes

**DOI:** 10.1186/1532-429X-15-S1-W15

**Published:** 2013-01-30

**Authors:** L Szczepaniak, MD Nelson, L Smith, EW Szczepaniak

**Affiliations:** 1 Cedars-Sinai Medical Center, Los Angeles, CA, USA

## Background

The risk of heart failure in obesity and type 2 diabetes is greater than can be accounted for by hypertension and coronary artery disease. Rodent studies indicate that in obesity and type 2 diabetes, lipid over-storage in cardiac myocytes produces lipotoxic intermediates leading to heart failure. Cardiac steatosis was previously demonstrated in explanted hearts of patients with end-stage nonischemic cardiomyopathy. We demonstrated that cardiac steatosis precedes the onset of cardiomyopathy in individuals with impaired glucose tolerance or in patients with type 2 diabetes mellitus using 1.5 T MR system. To perform cardiac MR Spectroscopy studies using 3 Tesla MRI system we had to overcome the particular challenges at high magnetic field.

## Methods

Localized spectroscopy can distinguish between triglyceride droplets localized in the cytosol of cardiomyocytes (i.e. an aqueous microenvironment) and triglycerides stored in adipocytes (i.e. a lipid microenvironment). During 1H-MRS, these different microenvironments cause triglycerides to resonate at different frequencies. In this study to determine myocardial triglyceride content we used a 3-Tesla Verio whole-body system (Siemens) equipped with spectroscopy and cardiac packages allowing for compensation of respiratory motion (PACE) and cardiac motion ( Siemens Work in Progress package). We positioned the spectroscopic volume of interest within bi-ventricular septum using the end-systolic cine images in 3 planes, collected as patients held their breath at the end of expiration. During acquisition of spectroscopic data, patients breathed freely. The spectroscopic signal was acquired with cardiac triggering at the end of systole and with respiratory gating at end expiration. We used the PRESS sequence (Point-RESolved Spectroscopy) for spatial localization, and the interpulse delay was defined by the length of a respiratory cycle (≈4 seconds).

## Results

After careful setup to compensate and to synchronize respiratory and cardiac motion we were able to collect high quality cardiac magnetic resonance spectra at 3 Tesla in humans in vivo. We recognized advantages of high field such as: 1) enhanced spectral intensity allowing using smaller volume of interest and shorter period for data collection; and 2) better spatial resolution of resonances. However, for a successful data collection the motion compensation had to be nearly perfect. This requirement sometimes presents an enormous challenge.

## Conclusions

Cardiac spectroscopy at 3 Tesla presents usual advantages such as enhanced spectral intensity and spatial resolution of resonances. However, it has zero-tolerance to motion.

## Funding

Lincy Foundation

